# Pathologic characteristics of histiocytic and dendritic cell neoplasms

**DOI:** 10.1007/s44313-024-00015-9

**Published:** 2024-05-07

**Authors:** Sun Och Yoon

**Affiliations:** Department of Pathology, Yonsei University College of Medicine, Severance Hospital, 50-1 Yonsei-Ro, Seodaemun-Gu, Seoul, 03722 South Korea

**Keywords:** Mononuclear phagocyte system, Histiocytic and dendritic cell neoplasms, Histiocytosis, Immunophenotyping, Molecular genetics

## Abstract

Histiocytic and dendritic cell neoplasms comprise diverse tumors originating from the mononuclear phagocytic system, which includes monocytes, macrophages, and dendritic cells. The 5th edition of the World Health Organization (WHO) classification updating the categorization of these tumors, reflecting a deeper understanding of their pathogenesis.

In this updated classification system, tumors are categorized as Langerhans cell and other dendritic cell neoplasms, histiocyte/macrophage neoplasms, and plasmacytoid dendritic cell neoplasms. Follicular dendritic cell neoplasms are classified as mesenchymal dendritic cell neoplasms within the stroma-derived neoplasms of lymphoid tissues.

Each subtype of histiocytic and dendritic cell neoplasms exhibits distinct morphological characteristics. They also show a characteristic immunophenotypic profile marked by various markers such as CD1a, CD207/langerin, S100, CD68, CD163, CD4, CD123, CD21, CD23, CD35, and ALK, and hematolymphoid markers such as CD45 and CD43. In situ hybridization for EBV-encoded small RNA (EBER) identifies a particular subtype. Immunoprofiling plays a critical role in determining the cell of origin and identifying the specific subtype of tumors. There are frequent genomic alterations in these neoplasms, especially in the mitogen-activated protein kinase pathway, including *BRAF* (notably *BRAF* V600E), *MAP2K1*, *KRAS*, and *NRAS* mutations, and *ALK* gene translocation.

This review aims to offer a comprehensive and updated overview of histiocytic and dendritic cell neoplasms, focusing on their ontogeny, morphological aspects, immunophenotypic profiles, and molecular genetics. This comprehensive approach is essential for accurately differentiating and classifying neoplasms according to the updated WHO classification.

## Introduction

Histiocytic and dendritic cell neoplasms represent a unique and complex group of disorders characterized by the proliferation of cells derived from the mononuclear phagocyte system [1–6].

Recently, there have been significant advancements in understanding “histiocytosis,” a rare and clinically heterogeneous disorder. It is characterized by the abnormal infiltration of monocytes, macrophages, or dendritic cells into various organs and tissues, including the skin, lymph nodes, lungs, bones, central nervous system, and heart. The clinical symptoms of histiocytosis can manifest as tumor mass effects, such as compression, inflammation leading to progressive fibrosis, and generalized symptoms, including fever, weight loss, and fatigue. Patients may also experience organ-specific manifestations or a combination of these symptoms [4–6].

Histiocytic and dendritic cell tumors, including Langerhans cell histiocytosis (LCH), Erdheim–Chester disease (ECD), juvenile xanthogranuloma (JXG), and Rosai–Dorfman disease (RDD), are characterized by intense inflammatory infiltration. This infiltration contains macrophages, lymphocytes, eosinophils, plasma cells, multinucleated giant cells, and less commonly, neutrophils. They also exhibit neoplastic features, such as mutations in the mitogen-activated protein kinase (MAPK) pathway. However, these tumors are not typically hyperproliferative, distinguishing them from more aggressive malignancies such as histiocytic sarcoma. In many cases, it is challenging to differentiate these lesions from fibroinflammatory processes during pathological diagnosis because of their overlapping characteristics in both inflammatory and neoplastic conditions [4–6].

With the expanded knowledge, histiocytosis has been systematically classified based on its characteristic histopathology, immunophenotypes, molecular genetics, and clinical features. The categories included the Langerhans-related group (L group), cutaneous and mucocutaneous group (C group), Rosai–Dorfman disease (R group), malignant histiocytosis group (M group), and a group comprising hemophagocytic lymphohistiocytosis and macrophage activation syndrome (H group) [4–6]. In alignment with these advancements, the WHO classification of histiocytic and dendritic cell neoplasms has evolved [1–3]. The classification system is primarily based on the cell of origin, which is important for understanding these disorders.

Morphologically, these neoplasms exhibit a range of histopathological features that are critical for identification. Immunohistochemistry is a fundamental tool for the diagnosis of these disorders and provides detailed insights into the expression patterns of specific markers associated with different neoplasm subtypes. Furthermore, advancements in molecular biology have elucidated the genetic and molecular mechanisms underlying these neoplasms, revealing mutations and alterations that contribute to their pathogenesis and offer potential targets for novel therapeutic interventions [1–6]. Therefore, this review aims to elucidate the intricate nature of these neoplasms and offer a comprehensive overview of their cell ontogeny, morphological characteristics, immunophenotypic features, and molecular aspects.

## Overview of the cell ontogeny

Histiocytic and dendritic cell neoplasms originate from the mononuclear phagocytes. This system includes monocytes, macrophages, and dendritic cells, which are essential sources of neoplasms. Mononuclear phagocytes function as specialized cells in the immune system. Monocytes can differentiate into diverse cell types, including macrophages and DCs, within the mononuclear phagocytic system. Dendritic can be classified into two main types: conventional DCs (cDC) and plasmacytoid DCs (pDC). Traditionally, macrophages have been regarded as phagocytes and dendritic cells as antigen-presenting cells. However, these cell types exhibit significant overlap in their properties and functions within the immune response. The term “histiocyte,” though not an actual cell type in the mononuclear phagocyte system, is often used to refer to macrophages or to encompass both macrophages and dendritic cells. In pathology, the terms “histiocyte” or “histiocytosis” are used diagnostically to imply that the disease originates from cells of the mononuclear phagocyte system [3, 5–13].

**Fig. 1:**
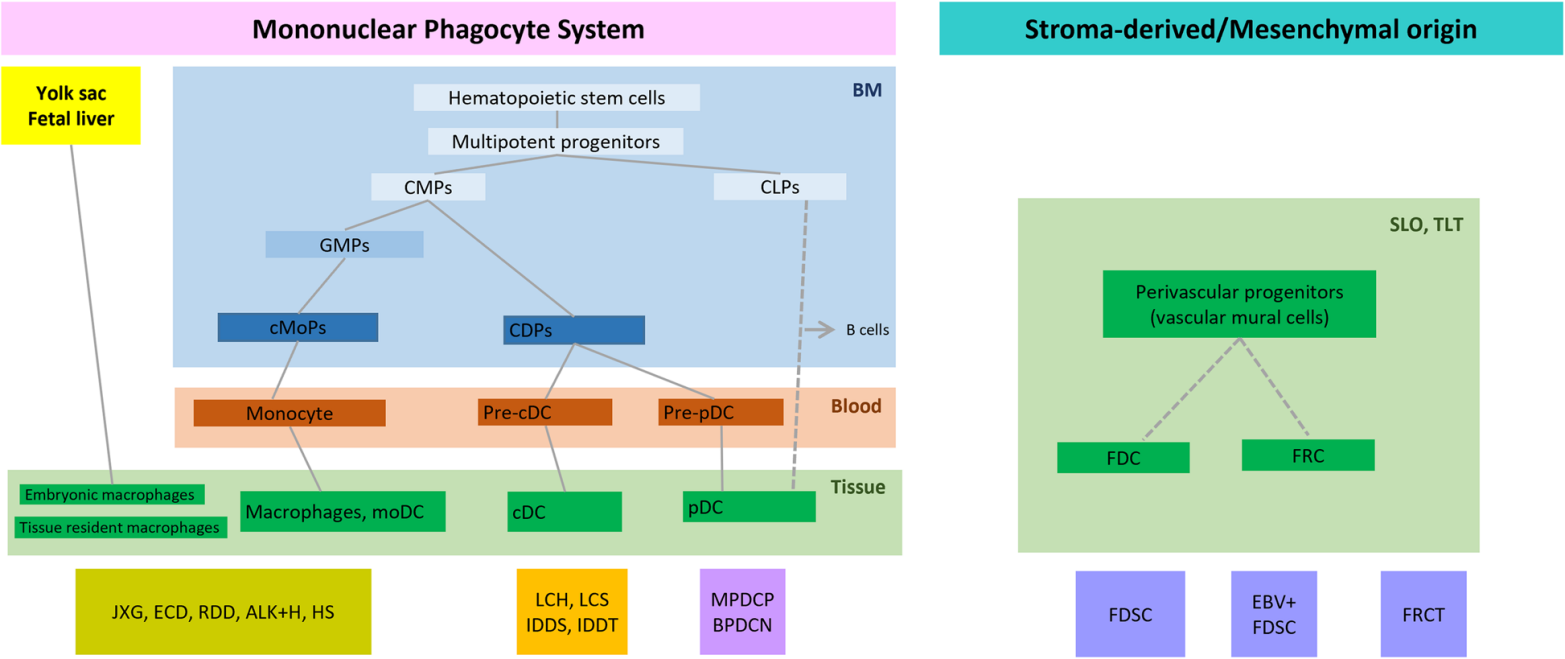
Overview of the origin and development of various cell types within the mononuclear phagocyte system, as well as those of stroma-derived/mesenchymal origin in lymphoid tissues, alongside their neoplastic counterparts. Abbreviations: BM, bone marrow; CMPs, common myeloid progenitors; CLPs, common lymphoid progenitors; GMPs, granulocyte–macrophage progenitors; cMoPs, common monocyte progenitors; CDPs, common dendritic cell progenitors; cDC, conventional dendritic cells; pDCs, plasmacytoid dendritic cells; moDC, monocyte-derived dendritic cell; JXG, juvenile xanthogranuloma; ECD, Erdheim-Chester disease; RDD, Rosai-Dorfman disease; ALK + H, ALK-positive histiocytosis; HS, histiocytic sarcoma; LCH, Langerhans cell histiocytosis; LCS, Langerhans cell sarcoma; IDDS, interdigitating dendritic cell sarcoma; IDDT, indeterminate dendritic cell tumor; MPDCP, mature plasmacytoid dendritic cell proliferation associated with myeloid neoplasm; BPDCN, blastic plasmacytoid dendritic cell neoplasm; SLO, secondary lymphoid organ; TLT, tertiary lymphoid tissue; FDC, follicular dendritic cell; FRC, fibroblastic reticular cell; FDCS, follicular dendritic cell sarcoma; EBV, Epstein Barr virus; FRCT, fibroblastic reticular cell tumor

Regarding the pathological characteristics of histiocytic and dendritic cell neoplasms, categories and subtypes are divided according to cell ontogeny, referring to the origin and development of various cell types in the mononuclear phagocyte system. The following provides an understanding of the origin and development of different cell types. This overview focuses on several key cell types and details their origins, typical immunophenotypes, and possible neoplastic counterparts (Fig. 1).

### Monocytes

Monocytes are the circulating immune cells that release cytokines during inflammation. They can enter tissues and differentiate into effector cells including macrophages and dendritic cells. Monocytes originate from hematopoietic stem cells in the bone marrow and differentiate first into common monocyte progenitors and then into monocytes. The complete pathway is as follows: hematopoietic stem cells (HSCs) → multipotent progenitors → common myeloid progenitors (CMPs) → granulocyte–macrophage progenitors (GMPs) → common monocyte progenitors (cMoPs) → monocytes (Fig. 1). The neoplastic counterpart of monocytes is acute monocytic leukemia, characterized by rapid and uncontrolled proliferation of monocytes [3, 5, 7–13].

### Macrophages

Macrophages are found in almost all body tissues. They play crucial roles in organ development, homeostasis, immune response, and tissue repair. Macrophages originate from HSCs in bone marrow. This pathway typically involves the differentiation of HSCs to CMPs, then to GMPs, and subsequently to cMoPs. cMoPs differentiate into monocytes, which circulate in the bloodstream and eventually migrate into tissues to become macrophages. Additionally, some tissue-resident macrophages are derived from embryonic precursors. These cells, known as yolk sac-derived macrophages or fetal liver progenitors, can colonize certain tissues during early development. Unlike bone marrow-derived macrophages, embryonic macrophages can self-renew within tissues and maintain their populations independently of hematopoietic stem cells from the bone marrow (Fig. 1). Macrophages express markers, such as CD45, CD4, CD68, CD163, and lysozyme. Conditions such as histiocytic sarcoma, Rosai-Dorfman disease, ALK-positive histiocytosis, ECD, and juvenile xanthogranuloma are neoplastic counterparts of macrophages [3, 7–13].

### Dendritic cells

The primary function of conventional dendritic cells (cDC) is to process and present antigens to T cells, effectively bridging innate and adaptive immune responses. cDCs originating from HSCs in bone marrow. The typical pathway is HSCs → multipotent progenitors → CMPs → common dendritic cell progenitors (CDPs) → cDC. These include Langerhans cells (expressing S100 protein, CD1a, CD207, and CD4), indeterminate dendritic cells (positive for S100 protein, CD1a, and CD4, but negative for CD207), interdigitating dendritic cells (positive for S100 protein and CD4, but negative for CD1a and CD207), and interstitial dendritic cells (positive for fascin, factor XIIIa, and occasionally positive for CD4). Langerhans cells (LCs) are located in the epidermis and mucosal lining of the bronchial epithelium. When activated by various stimuli, LCs migrate to draining lymph nodes and differentiate into interdigitating dendritic cells. LCH, Langerhans cell sarcoma, interdigitating dendritic cell sarcoma, and indeterminate dendritic cell tumor are among the neoplastic counterparts [3, 5, 7–14].

### Plasmacytoid dendritic cells

Plasmacytoid dendritic cells (pDCs) play a significant role in antiviral immunity and have been implicated in systemic autoimmunity. They are known for their ability to produce large amounts of type I interferons in response to viral infections, which are crucial for antiviral immune responses. Furthermore, their role in systemic autoimmunity has been recognized, particularly in conditions such as systemic lupus erythematosus. Initially, pDCs were believed to originate from CMPs through CDPs. However, recent studies have suggested a more complex mechanism. Some studies have indicated that pDCs may develop from lymphoid progenitors or progenitors shared with certain lymphocytes, such as B cells (Fig. 1). They are characterized by the expression of CD123 (IL-3R), CD303 (BDCA2), CD304 (BDCA4), and TCF4. The neoplastic counterparts of pDCs are mature plasmacytoid dendritic cell proliferation associated with myeloid neoplasm and blastic plasmacytoid dendritic cell neoplasm [3, 15, 16].

### Follicular dendritic cells

Follicular dendritic cells (FDCs) play a crucial role in adaptive immunity. They contribute to the formation of germinal centers and present antigens to B cells. Additionally, they play a pivotal role in the activation and maturation of B cells, leading to the development of robust humoral immune responses. These cells originate from stromal or mesenchymal progenitors in lymphoid tissues, and not from the mononuclear phagocyte system. Within secondary lymphoid organs such as the lymph nodes and spleen, as well as in tertiary lymphoid tissues that form in non-lymphoid organs, FDCs are thought to be generated from perivascular precursors (Fig. 1). However, the details of their developmental lineages in the field of immunology remain to be elucidated. Follicular dendritic cells express markers such as CD21, CD23, CD35, clusterin, CXCL13, and D2-40 (podoplanin). The neoplastic counterpart of these cells is follicular dendritic cell sarcoma [3, 17–20].

### Fibroblastic reticular cells

Fibroblastic reticular cells (FRCs) are specialized cells found within lymphoid organs, such as the lymph nodes and spleen. They play several key roles in the immune system, including providing structural support to the lymphoid stroma, regulating the movement and positioning of immune cells (such as T and B cells) within these organs, and modulating immune responses. These cells originate from stromal or mesenchymal precursors located in the perivascular spaces of the lymphoid organs and tissues. FRCs share a common precursor with FDCs located around the walls of blood vessels (Fig. 1). They are characterized by the expression of smooth muscle actin and desmin. The fibroblastic reticular cell tumor is thought to be a neoplasm originating from putative stromal fibroblastic reticular cells [3, 20–22].

## Morphological characteristics

**Fig. 2:**
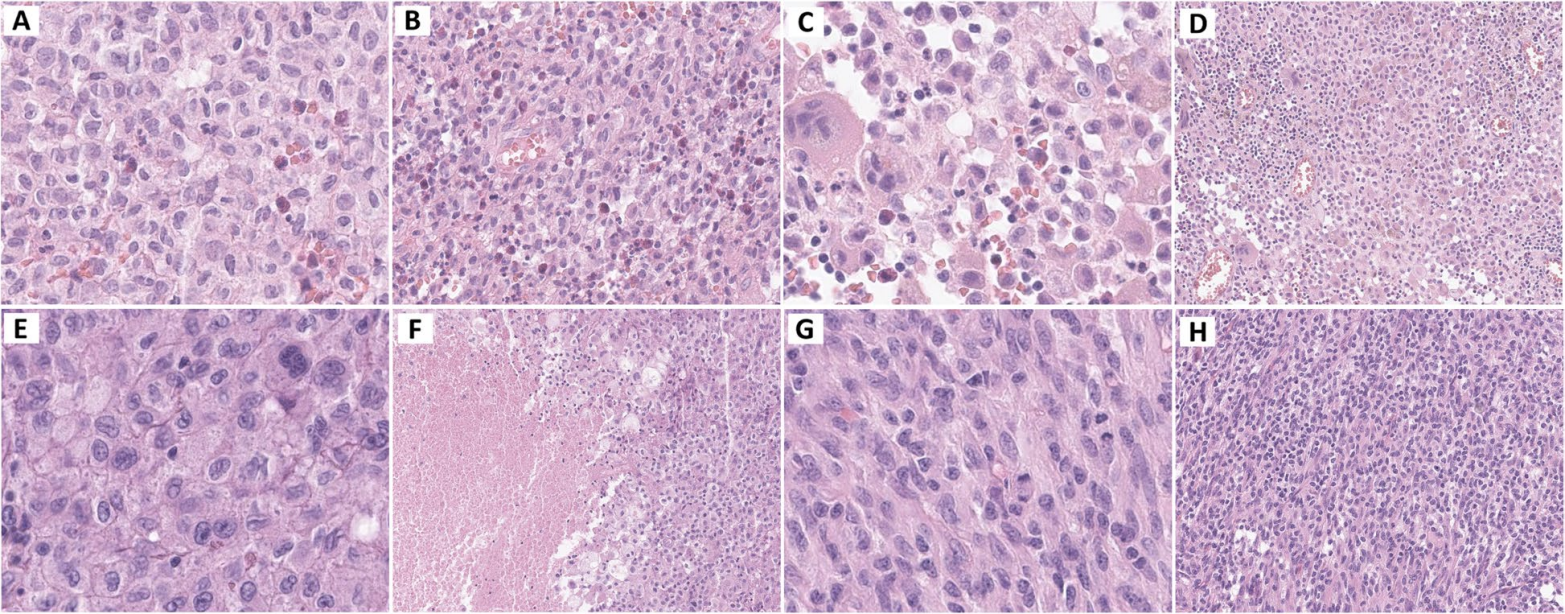
Tumor cells of LCH are round-to-oval histiocytes, with characteristic nuclear grooves and convolution, and prominent eosinophil infiltrates noted within the tumor microenvironment (**A** and **B**). In another LCH, tumor cells showing nuclear grooves are admixed with multinucleated giant cells of the osteoclast type, foamy histiocytes, and numerous small lymphocytes (**C** and **D**). In LCS, high-grade cytologic pleomorphism and confluent tumor necrosis are observed (**E** and **F**). In IDDS, the tumor cells appear as spindle to epithelioid cells, with a fascicular growth pattern, interspersed with small lymphocytes (**G** and **H**). LCH, Langerhans cell histiocytosis; LCS, Langerhans cell sarcoma; IDDS, interdigitating dendritic cell sarcoma

**Fig. 3:**
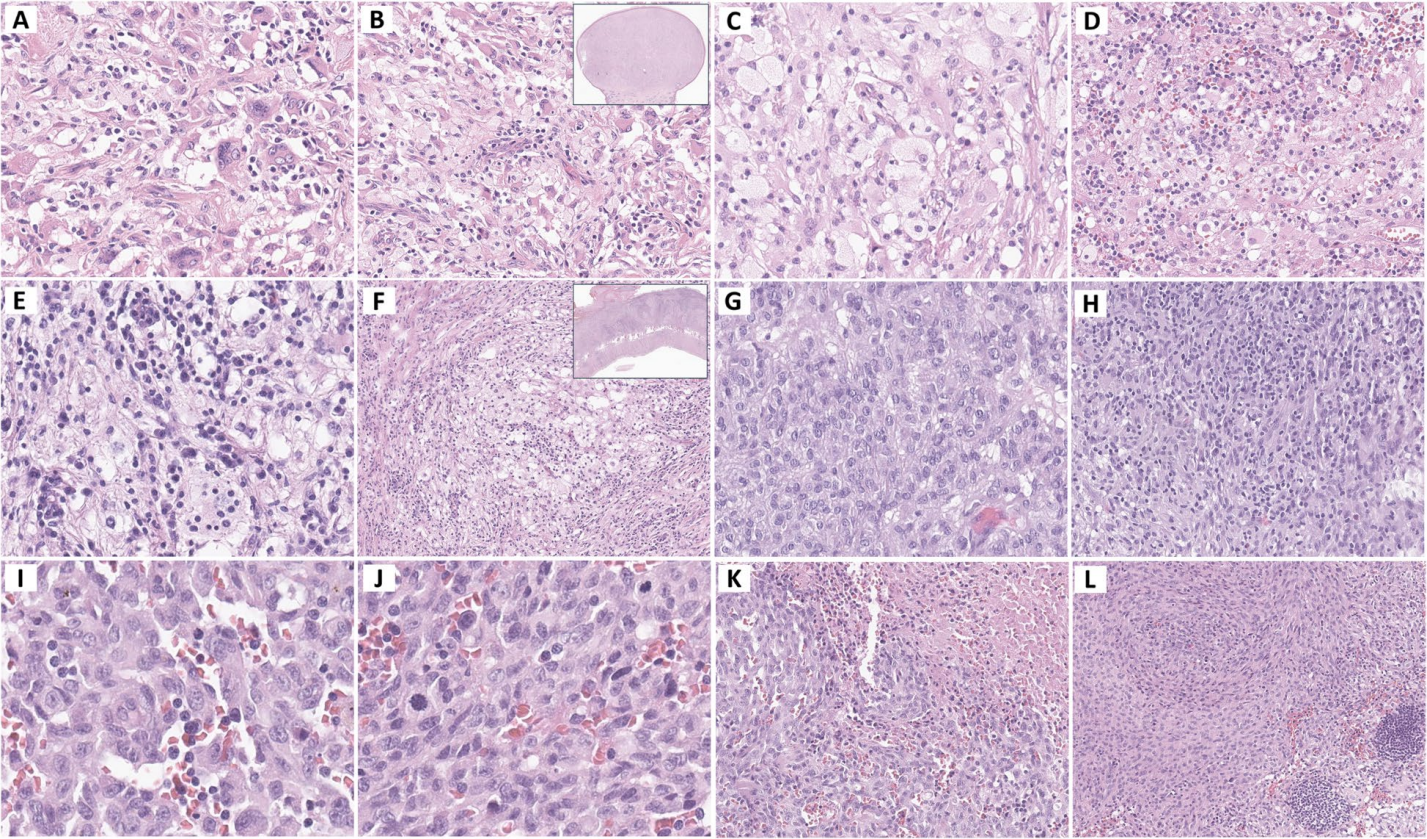
JXG (**A** and **B**), noted as a well-circumscribed lesion (inset, **B**), consists of foamy histiocytes and Touton giant cells, mixed with lymphocytes. In ECD (**C** and **D**), a collection of foamy histiocytes intermingled with lymphocytes is observed. In RDD (**E** and **F**), prominent foamy histiocytes exhibiting emperipolesis are identified, accompanied by extensive plasma cell infiltration. At low magnification, the histiocytic clusters and lymphoplasmacytic tumor microenvironment create a contrasting light and dark pattern (inset, **F**). In ALK-positive histiocytosis (**G** and **H**), oval to spindle cells exhibit growth in fascicular and storiform patterns, interspersed with lymphocytes. Lastly, in HS (**I**-**L**), large pleomorphic tumor cells show high-grade cytologic atypia with increased mitosis and confluent tumor necrosis, displaying a storiform or whirling pattern that displaces the normal architecture of the lymph node. Abbreviations: JXG, juvenile xanthogranuloma; ECD, Erdheim-Chester disease; RDD, Rosai-Dorfman disease; HS, histiocytic sarcoma

**Fig. 4:**
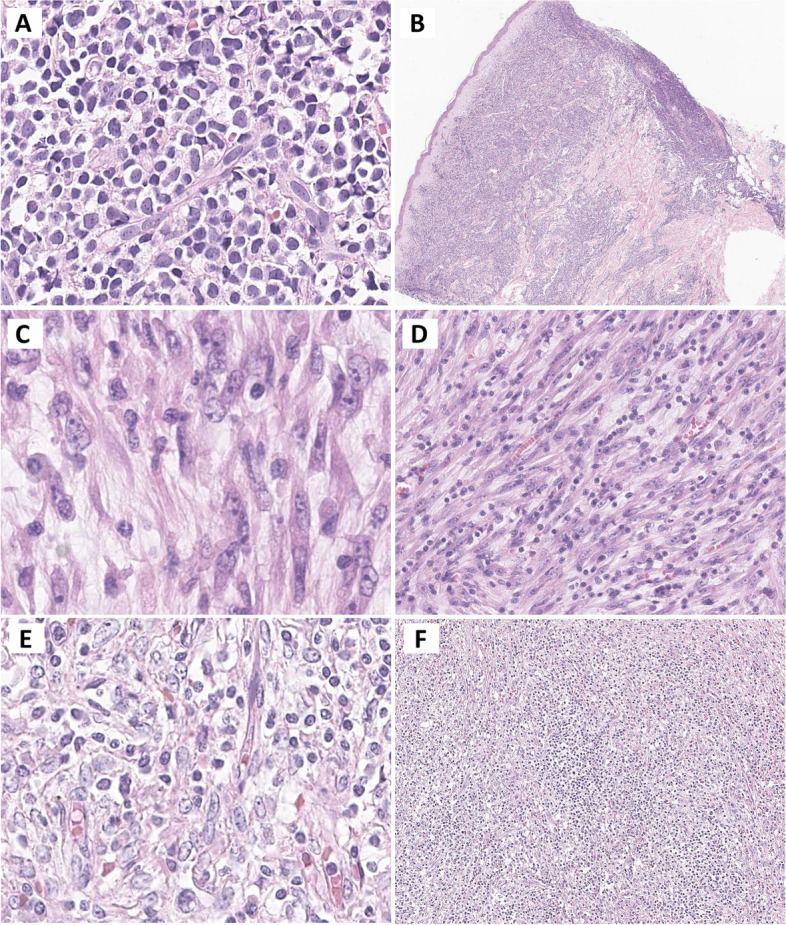
In BPDCN (**A** and **B**), immature blastic tumor cells infiltrate the dermis and subcutis, while sparing the epidermis. In FDCS (**C** and **D**), spindled and ovoid tumor cells, occasionally showing binucleation, grow in a fascicular pattern, interspersed with small lymphocytes. In EBV + FDSC, spindle-to-oval cells, resembling follicular dendritic cells, are either dispersed or form loose whorled fascicles, admixed with a rich and prominent lymphoplasmacytic infiltrate (**E** and **F**). Abbreviations: BPDCN, blastic plasmacytoid dendritic cell neoplasm; FDCS, follicular dendritic cell sarcoma; EBV, Epstein–Barr virus; EBV + FDCS, EBV-positive inflammatory follicular dendritic cell sarcoma

Neoplasms arising from dendritic and monocyte/macrophage cells are rare and diverse and present a range of histopathological features. Generally, excisional biopsy is preferred over core biopsy or fine needle aspiration because of the complicated architecture, mixed inflammatory microenvironment, and uneven distribution of tumor cells within the lesions. The following description outlines the characteristics of these neoplasms and provides insights into their cellular and tissue morphologies. Understanding their distinctive histopathological features is critical for the accurate diagnosis of these rare but complex tumors. Table 1 and Figs. 2, 3 and 4 show the overall characteristics and representative images of each subtype.

**Table 1:** Overview of the characteristics of histiocytic and dendritic cell neoplasms

Subtype (ICD-O coding, WHO 5th)	Histomorphology	Immunophenotype	Molecular genetics
Langerhans cell and other dendritic cell neoplasms			
Langerhans cell histiocytosis (9751/1; 9751/3)	Tumor cells: Large, round-to-oval histiocytes; nuclei ranging from grooved to convoluted; moderately abundant, eosinophilic cytoplasm; minimal atypia TME: Inflammatory infiltrates; prominent eosinophils; lymphocytes; multinucleated giant cells; fibrosis in advanced stages	Positive expression : CD1a (membranous), S100 (nuclear and cytoplasmic), CD207/langerin (granular cytoplasmic), and CD68 (Golgi dot-like staining); S100 protein, CD163; *BRAF* V600E mutated protein (VE1)	Mutations in the MAPK pathway (~ 85%): *BRAF* V600E (50–60%), *MAP2K1 (20%), BRAF, ARAF, NRAS, KRAS, HRAS*
Langerhans cell sarcoma (9756/3)	Tumor cells: Pleomorphic histiocytes exhibiting high-grade cytology; large, atypical nuclei, clumped chromatin, high mitotic activity, frequent atypical mitoses, lacking nuclear grooves TME: Variable eosinophil infiltrates	Positive expression: CD1a, S100, CD207/langerin (variable to focal expression)	Limited information, Mutations in the MAPK pathway: *KRAS*, *BRAF* V600E (rare)
Indeterminate dendritic cell tumor (9757/3)	Tumor cells: Monomorphic, mononuclear cells resembling Langerhans cells; grooved nuclei, ample eosinophilic cytoplasm TME: A mixture of inflammatory cells including eosinophils and lymphocytes, reactive macrophages, multinucleated giant cells	Positive expression: CD1a, S-100 protein Negative expression: CD207/langerin	Limited information, *BRAF* V600E (rarely)
Interdigitating dendritic cell sarcoma (9757/3)	Tumor cells: Spindle-shaped to epithelioid cells; abundant cytoplasm, indistinct cell borders; occasional multinucleation, grooves, vesicular chromatin, distinct nucleoli; growth in sheets, fascicles, whorls, storiform pattern TME: Interspersed or aggregated small lymphocytes and plasma cells	Positive expression: S-100 protein (highlighting dendritic cell processes); one or more hematolymphoid markers of such as CD45, CD4, CD43 Negative expression: Langerhans cell markers (CD1a, CD207/langerin), follicular dendritic cell markers (CD21, CD23, CD35), other markers specific to certain neoplasms or melanoma	Mutations in the MAPK pathway: *BRAF* V600E, *KRAS, NRAS, MAP2K1*
Histiocyte/Macrophage neoplasms			
Juvenile xanthogranuloma (9749/1)	Tumor cells: Large, xanthomatous, foamy histiocytes, resembling dermal macrophages and lacking significant nuclear pleomorphism; growth in non-encapsulated, well-demarcated, and circumscribed pattern TME: Touton giant cells; a variable mixture of lymphocytes, eosinophils, plasma cells, neutrophils, and mast cells; fibrosis observed in regressed lesions	Positive expression: CD68, CD163, CD4, CD14, factor XIIIa, fascin; S100 protein (occasionally) Negative expression: CD1a, CD207langerin, ALK; BRAF-VE1(usually)	MAPK pathway: *MAPK21, NRAS, KRAS, CSF1R* mutations; *BRAF* fusion, *NTRK1* fusion
Erdheim-Chester disease (9749/3)	Tumor cells: Bland, foamy, lipid-laden, and/or small mononuclear histiocytes; occasionally, admixed with Langerhans cell histiocytosis TME: Touton giant cells, small lymphocytes, plasma cells, and/or neutrophils; fibrosis, which is sometimes prominent	Positive expression: CD163, CD68, CD14, CD4, factor XIIIa, fascin; BRAF-VE1; S100 protein (rarely) Negative expression: CD1a, CD207/langerin	Mutations in the MAPK pathway: *BRAF* V600E (50–60%), *ARAF, BRAF, NRAS, KRAS, MAP2K1; PIK3CA*
Rosai-Dorfman disease (9749/3)	Tumor cells: Large histiocytes; round to oval vesicular nuclei, prominent nucleoli; abundant pale cytoplasm with indistinct borders; emperipolesis, a hallmark feature; minimal atypia; low mitotic activity; massive, marked sinus expansion pattern in the lymph node TME: Abundant polyclonal plasma cells, with occasional increases in IgG4-positive plasma cells; Lymphocytes and, less commonly, neutrophils; rare eosinophils; capsular and stromal fibrosis	Positive expression; S100 (highlighting emperipolesis); OCT2, cyclinD1; macrophage markers (CD68, CD163); IgG4 (plasma cells) Negative expression: CD1a, CD207/langerin, ALK, BRAF-VE1(usually)	Mutations in the MAPK/ERK pathway: *KRAS, NRAS, MAP2K1, ARAF, CSF1R*, *BRAF* V600E (rarely)
ALK-positive histiocytosis (9750/3)	Tumor cells: Large oval, foamy, and spindle cell histiocytes in variable proportions, lacking high-grade cytologic atypia; large histiocytes; irregularly folded nuclei, fine chromatin, abundant eosinophilic cytoplasm, with occasional minor emperipolesis; foamy histiocytes showing vacuolated cytoplasm and irregularly folded nuclei, sometimes accompanied by Touton giant cells; spindle histiocytes exhibiting a fascicular or storiform growth pattern, elongated nuclei, and eosinophilic cytoplasm TME: Comprised of small lymphocytes and plasma cells	Positive expression: ALK (cytoplasmic, rarely membranous or Golgi dot pattern; never in nuclear pattern); at least two histiocytic markers (CD163, CD68, CD14, CD4, lysozyme); occasionally S100 protein, cyclinD1, OCT2 Negative expression: CD1a, CD207/langerin, CD30, BRAF-VE1	*ALK::KIF5B* (most common); *CLTC-ALK, TPM3-ALK, TFG-ALK, EML4-ALK,* and *DCTN1-ALK*
Histiocytic sarcoma (9755/3)	Tumor cells: Non-cohesive, large with abundant eosinophilic cytoplasm; variably pleomorphic, grooved, or irregularly folded nuclei, distinct nucleoli; prominent mitotic activity, atypical mitoses; diffuse sheets, sinusoidal distribution TME: Reactive inflammatory and stromal cells admixture	Positive expression: two or more histiocytic markers (CD163, CD68, lysozyme); CD4, CD45; S100 protein (usually patch) Negative expression: Langerhans cell markers (CD1a, CD207/langerin), follicular dendritic cell markers (CD21, CD23, CD35), myeloid cell markers (myeloperoxidase, CD13); ALK; melanocytic markers (SOX10, HMB-45); epithelial markers (cytokeratins); and other specific markers for certain neoplasms, including hematopoietic tumors	Mutations in the MAPK pathway: *KRAS, BRAF V600E, NRAS, MAP2K1*, and CSF1R
Plasmacytoid dendritic cell neoplasms			
Mature plasmacytoid dendritic cell proliferation associated with myeloid neoplasm (no code)	Tumor cells: Medium-sized, mature plasmacytoid cells; round or oval nuclei, moderate amphophilic cytoplasm; absence of mitotic figures; aggregation or interstitial scattering in bone marrow TME: Associated with underlying myeloid neoplasm	Positive expression: CD123; other pDC markers such as TCF4, TCL1, CD303, CD304 with possible aberrant loss; CD34, TdT, CD56 (absent, low, or partial expression)	Depends on associated myeloid neoplasm
Blastic plasmacytoid dendritic cell neoplasm (9727/3)	Tumor cells: Medium-sized, immature, and blastic, resembling lymphoblasts or myeloblasts; scanty cytoplasm, eccentrically positioned round or irregular nuclei, fine chromatin, and one to several inconspicuous nucleoli; presence of variable mitotic figures and necrosis	Immunophenotypic diagnostic criteria: 1. Mandatory expression of CD123 along with at least one other pDC marker (such as TCF4, TCL1, CD303, or CD304), in addition to either CD4 and/or CD56 Or 2. Expression of any three pDC markers (including CD123, TCF4, TCL1, CD303, CD304), along with the absence of all expected negative markers of lymphoid or myeloid lineage (such as CD3, CD14, CD19, CD34, lysozyme, and myeloperoxidase)	NF-κB pathway
Mesenchymal dendritic cell neoplasms of lymphoid tissues: Follicular dendritic cell neoplasms^a^			
Follicular dendritic cell sarcoma (9758/3)	Tumor cells: Spindled, ovoid, or epithelioid shape with moderate eosinophilic cytoplasm, indistinct cell borders; elongated nuclei with vesicular chromatin, thin nuclear membranes, distinct nucleoli, intranuclear pseudoinclusions, occasional binucleation; occasional prominent cytologic atypia; growth patterns in whorls, fascicles, syncytial sheets, storiform pattern TME: Interspersed small lymphocytes and plasma cells within the tumor or forming aggregates	Positive expression: Two or more FDC markers including CD21, CD23, CD35, clusterin, CXCL13, D2-40 (podoplanin) Negative expression: S100 protein; other specific markers for certain hematolymphoid neoplasms	Limited information, NF-kB pathway, *BRAF* V600E
EBV-positive inflammatory follicular dendritic cell sarcoma (9758/3)	Tumor cells: Spindle to oval shape with indistinct borders; scant to moderate cytoplasm; vesicular nuclei with small, distinct nucleoli; occasional binucleation; variable nuclear atypia and cellular pleomorphism; rare mitotic figures, often with necrosis and hemorrhage; growth in dispersed or loosely whorled fascicle patterns TME: Prominent lymphoplasmacytic infiltrate	Positive expression: FDC markers (CD21, CD23, CD35, CXCL13, D2-40); rarely, fibroblastic/myoid markers (SMA); EBV-encoded small RNA (EBER)	Limited information
Fibroblastic reticular cell tumor (9759/3)	Tumor cells: Spindle to ovoid shape with indistinct cell borders and vesicular nuclei; growth patterns in whorls, fascicles, or sheets; variable nuclear pleomorphism; overlapping morphological features with follicular dendritic cell sarcoma and interdigitating dendritic cell sarcoma; presence of intercellular collagen fibrils TME: interspersed with small lymphocytes	Positive expression: one of more of the following markers (cytokeratin, actin, desmin, with delicate cell processes pattern) Negative expression: FDC markers (CD21, CD23, CD35, CXCL13); interdigitating dendritic cell markers (S100)	Limited information

^a^Follicular dendritic cell neoplasms are classified as mesenchymal dendritic cell neoplasms within the stroma-derived neoplasms of lymphoid tissues rather than histiocytic and dendritic cell neoplasms in the 5th WHO classification

References are displayed in the main text section

Abbreviations: FDC, follicular dendritic cell; TME, tumor microenvironment

### Langerhans cell and other dendritic cell neoplasms

#### Langerhans cell histiocytosis

Tumor cells in LCH, often referred to as Langerhans cells, are typically large and oval or round. These cells are notable for their grooved-to-convoluted nuclei. The cytoplasm was usually eosinophilic and moderately abundant. These features are histologically distinctive and can aid in the diagnosis of LCH. The tumor cells display fine chromatin, a delicate nuclear membrane, inconspicuous nucleoli, and minimal atypia; however, mitoses may be frequent. The tumor microenvironment (TME) in LCH predominantly consists of eosinophils, lymphocytes, and occasionally multinucleated giant cells. Eosinophils are often a prominent feature, which is a notable aspect of LCH. Neutrophils and plasma cells are present but were generally less common (Fig. 2A-D). As the disease progresses or responds to treatment, the number of LCH cells may decrease and fibrosis may increase. In cases of central nervous system Langerhans cell histiocytosis (CNS-LCH), LCH-associated neurodegenerative diseases (LCH-ND) progressively develop in up to 10% of patients. In these cases, inflammatory infiltrates have been described, composed of foamy macrophages, CD1a + cells, and perivascular infiltrates of mononuclear cells expressing the *BRAF* V600E protein, along with neurodegeneration or demyelination [1–3, 5, 6, 23–26].

#### Langerhans cell sarcoma

Langerhans cell sarcoma (LCS) was characterized by pleomorphic histiocytes with high-grade cytology, indicating an aggressive tumor (Fig. 2E and F). This tumor can occur either as a primary de novo LCS or as a secondary LCS, with progression from low-grade LCH or transdifferentiation from other types of hematolymphoid neoplasms, such as follicular lymphoma or chronic lymphocytic leukemia. Tumor cells have large, atypical nuclei that are often plump or elongated, with clumped chromatin, high mitotic activity, and frequent atypical mitoses. Unlike LCH, LCS typically lacks characteristic nuclear grooves and shows variable eosinophil infiltrates [1–3, 5, 27, 28].

#### Indeterminate dendritic cell tumor

Indeterminate dendritic cell tumors (IDCT) are characterized by the proliferation of mononuclear histiocytes resembling Langerhans cells. These cells feature grooved nuclei and ample eosinophilic cytoplasm and express dendritic cell markers such as CD1a and S100. However, they lack Birbeck granules and do not express CD207/langerin. In skin lesions, which typically manifest as generalized skin eruptions, tumor cells infiltrate the dermis and/or subcutis, while sparing the epidermis. Inflammatory infiltrates were observed in the TME. The mixed inflammatory cells include eosinophils and lymphocytes, as well as reactive macrophages, and multinuclear giant cells [2, 3, 29, 30].

#### Interdigitating dendritic cell sarcoma

Interdigitating dendritic cell sarcoma (IDCS) is characterized by the proliferation of pleomorphic spindle-to-epithelioid cells showing differentiation of interdigitating dendritic cells. The tumor cells display abundant eosinophilic cytoplasm and indistinct borders. Their nuclei range from spindle-to-ovoid shaped and may show occasional multinucleation, grooves, vesicular chromatin, and distinct nucleoli. IDCS grows in patterns of sheets, fascicles, whorls, or storiform patterns (Fig. 2G and H). In the TME, small lymphocytes and plasma cells are interspersed or aggregated throughout the tumor [1–3, 31].

### Histiocyte/macrophage neoplasms

#### Juvenile xanthogranuloma

Juvenile xanthogranuloma (JXG) typically manifests as non-encapsulated, well-demarcated, and localized lesions on the skin and/or mucosal surfaces. Extracutaneous or disseminated JXG has also been identified. In such cases, it may be classified into the ECD category of the L group, particularly when accompanied by MAPK-activating mutations, or classified as ALK-positive histiocytosis when carrying ALK immunoreactivity and/or ALK gene rearrangement. JXG lesions are composed of large, xanthomatous, and foamy histiocytes resembling dermal macrophages that do not show significant nuclear pleomorphism. Characteristic features included Touton giant cells, which are multinucleated histiocytes distinguished by their unique arrangement of nuclei and cytoplasm (Fig. 3A and B). JXG lesions often contain a combination of lymphocytes, eosinophils, plasma cells, neutrophils, and mast cells. Fibrosis may increase later, corresponding to the regression of the lesion [1–3, 5, 6, 32, 33].

#### Erdheim-Chester disease

ECD is a unique type of non-Langerhans cell histiocytosis, primarily characterized by multi-organ infiltration with distinctive histiocytes. Widespread involvement can result in various clinical manifestations. Therefore, bilateral symmetric involvement of the long bones, detected in almost all patients, is a hallmark of ECD. Additionally, cardiopulmonary involvement is frequent and infiltration of the perinephric, periaortic, CNS, and skin regions represent other characteristic clinical features. Diagnosis is based on these clinical and radiological features complemented by ECD histology. The histiocytes in ECD are bland, foamy, and lipid-laden and are often accompanied by small mononuclear histiocytes. The tumor microenvironment frequently contains giant Touton cells, along with small lymphocytes, plasma cells, and neutrophils. Such histology can resemble xanthogranuloma, making differentiation between these conditions challenging based solely on histological examination (Fig. 3C and D). Fibrosis is a common and sometimes predominant feature of ECD, potentially leading to the misinterpretation of the lesion as a reactive fibroinflammatory process rather than as a neoplastic condition. LCH may be admixed on the same biopsy [1–3, 5, 6, 34].

#### Rosai-Dorfman disease

Rosai–Dorfman disease (RDD) is characterized by large histiocytes with round nuclei, prominent nucleoli, and abundant pale cytoplasm. Pleomorphisms, mitoses, and multinucleation were rarely observed. A notable feature of RDD is the marked sinus expansion in the lymph nodes, which is a key diagnostic criterion. Another hallmark is emperipolesis, which refers to the presence of intact hematopoietic cells within the cytoplasm of histiocytes. These cells include lymphocytes, plasma cells, neutrophils, and erythrocytes. In RDD, the tumor microenvironment is characterized by an abundance of polyclonal plasma cells accompanied by lymphocytes, neutrophils, and occasionally neutrophilic microabscesses. Capsular and stromal fibrosis, often extending into the perinodal soft tissue, is a characteristic of RDD. RDD frequently features an increased number of IgG4-positive plasma cells; therefore, differential diagnosis of IgG4-related disease is sometimes necessary. In cases of extranodal RDD, such as those involving the skin, bone, CNS, and head and neck, histiocytic clusters and a lymphoplasmacytic microenvironment created a light and dark pattern at low magnification (Fig. 3E and F). Emperipolesis can be subtle and a mix of foamy histiocytes and stromal fibrosis is often present, making diagnosis difficult. The presence of perivascular plasma cells is a helpful diagnostic indicator for extranodal RDD [1–3, 5, 6, 35, 36].

#### ALK-positive histiocytosis

ALK-positive histiocytosis is a relatively rare subtype of istiocytosis, which are rare disorders characterized by the accumulation of histiocytes in various tissues. The histological features of this condition vary among cases. Its histopathology is characterized by large, oval, foamy, and spindle-cell histiocytes in variable proportions. Large oval histiocytes displayed irregularly folded nuclei and abundant eosinophilic cytoplasm. Multinucleation and emperipolesis are occasionally observed. Foamy histiocytes showed vacuolated cytoplasm with irregularly folded nuclei, sometimes admixed with Touton giant cells. Spindle histiocytes are characterized by fascicular or storiform growth patterns, elongated nuclei, and eosinophilic cytoplasm. As a diagnostic criterion, high-grade cytological atypia, including features such as pleomorphisms and mitoses, was lacking in the histiocytes (Fig. 3G and H). The tumor microenvironment predominantly comprises small lymphocytes and plasma cells [3, 6, 33, 37].

#### Histiocytic sarcoma

Histiocytic sarcoma is characterized by non-cohesive large cells with abundant eosinophilic cytoplasm, which is a hallmark of this tumor. For the definition of malignant histiocytes belonging to the M group, cytological atypia and prominent mitotic activity, including atypical mitoses, are considered critical diagnostic criteria. These cells grew in patterns of diffuse sheets with a sinusoidal distribution. The nuclei of the tumor cells were oval, grooved, and irregularly folded, featuring vesicular chromatin and distinct nucleoli (Fig. 3I-L). Variable numbers of reactive inflammatory and stromal cells are often admixed [1–3, 5].

### Plasmacytoid dendritic cell neoplasms

#### Mature plasmacytoid dendritic cell proliferation associated with myeloid neoplasm

Mature plasmacytoid dendritic cell proliferation associated with myeloid neoplasms (MPDCP) has been newly categorized in the 5th edition of the World Health Organization (WHO) classification, following recent studies that have demonstrated the clonal proliferation and neoplastic nature of plasmacytoid dendritic cells (pDCs). MPDCP is characterized by the accumulation of mature cells that exhibit a plasmacytoid morphology and is associated with an underlying myeloid neoplasm. Chronic myelomonocytic leukemia (CMML) is the most frequently associated underlying myeloid neoplasm, followed by acute myeloid leukemia (AML), myelodysplastic neoplasms, and myeloproliferative neoplasms. MPDCP is present in approximately 5% of the AML cases. These cells displayed round or oval nuclei with moderate amounts of amphophilic cytoplasm. A notable feature was the absence of mitotic figures, suggesting that the cells were not actively dividing. This characteristic aligns with the “mature” aspect of these cells. Within the bone marrow, MPDCP cells may be aggregated or interstitially scattered [3, 38–40].

#### Blastic plasmacytoid dendritic cell neoplasm

Blastic plasmacytoid dendritic cell neoplasms (BPDCN) were newly classified within the category of histiocytic and dendritic cell neoplasms in the 5th edition of the WHO classification. This reclassification follows the recent concept that these tumors are derived from pDCs. BPDCN is characterized by the presence of immature blastic cells that exhibit plasmacytoid dendritic cell differentiation. Tumor cells exhibited cytological features of medium-sized immature blastic cells with scanty cytoplasm, eccentric round or irregular nuclei, fine chromatin, and one to several inconspicuous nucleoli. Mitotic figures vary and necrosis may be present. In the skin, which is one of the most commonly involved sites, the neoplastic infiltrate was typically centered in the dermis and extended to the subcutaneous tissue while sparing the epidermis and adnexal structures (Fig. 4A and B). In the lymph nodes, BPDCN predominantly involves the medullary and interfollicular areas. In the bone marrow, tumor cells infiltrate in diffuse and/or interstitial patterns [1–3, 41].

### Mesenchymal dendritic cell neoplasms of lymphoid tissues: Follicular dendritic cell neoplasms

#### Follicular dendritic cell sarcoma

In follicular dendritic cell sarcoma (FDCS), tumor cells exhibit a spindle, ovoid, or epithelioid morphology, characterized by moderate amounts of eosinophilic cytoplasm and indistinct cell borders, contributing to their typical syncytial appearance. The nuclei were elongated with vesicular chromatin, thin nuclear membranes, and distinct nucleoli. Binucleated cells reminiscent of follicular dendritic cells may be present. The tumor cells grow in whorls, fascicles, syncytial sheets, and storiform arrangements. Occasionally, tumor cells exhibited prominent cytologic atypia characterized by multinucleation and pleomorphic nuclei (Fig. 4C and D). In TME, small lymphocytes and plasma cells are interspersed within the tumor or form aggregates [1–3, 42].

#### EBV-positive inflammatory follicular dendritic cell sarcoma

EBV-positive inflammatory follicular dendritic cell sarcoma (EBV + inflammatory FDCS) occurs almost exclusively in the liver and spleen. This tumor is characterized by the proliferation of spindle-shaped to oval cells, which show follicular dendritic cell differentiation, along with a rich lymphoplasmacytic infiltrate and a consistent association with EBV. The tumor cells displayed indistinct cell borders, scanty to moderate amounts of cytoplasm, and vesicular nuclei with small, centrally located, and distinct nucleoli. Binucleated cells were also observed. The nuclear atypia varied significantly. Typically, bland-looking spindle cells are admixed with overtly pleomorphic cells that exhibit enlarged, irregularly folded, or hyperchromatic nuclei. Occasionally, tumor cells mimicked Hodgkin Reed-Sternberg cells (Fig. 4E and F). Mitotic figures were rare, and necrosis and hemorrhage were often observed. Tumor cells are either dispersed or form loose, whorled fascicles within the tumor microenvironment, which are marked by a prominent lymphoplasmacytic infiltrate [1–3, 43].

#### Fibroblastic reticular cell tumor

Fibroblastic reticular cell tumors (FRCTs) consist of spindle-to-ovoid cells arranged in whorls, fascicles, or sheets with interspersed small lymphocytes. Nuclear pleomorphism varies and multinucleation may occur. FRCT shows overlapping morphological features with follicular dendritic cell sarcoma and interdigitating dendritic cell sarcoma, with spindle-to-oval cell shapes, indistinct cell borders, and vesicular nuclei. Although not pathognomonic, the presence of intercellular collagen fibrils is characteristic [1–3, 44].

## Immunophenotypic characteristics

**Fig. 5:**
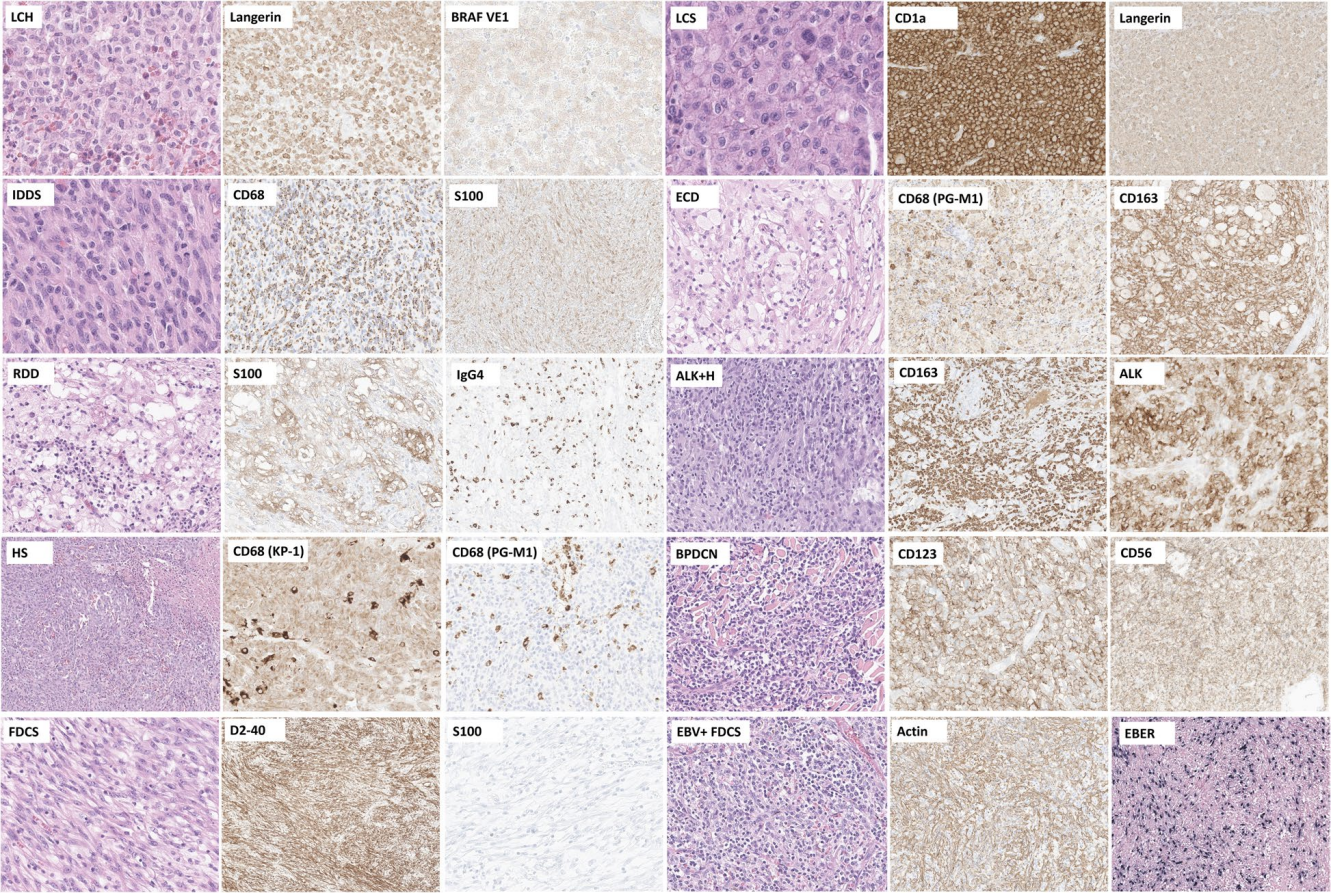
Representative images of immunohistochemical expression in each subtype. Abbreviations: LCH, Langerhans cell histiocytosis; LCS, Langerhans cell sarcoma; IDDS, interdigitating dendritic cell sarcoma; ECD, Erdheim-Chester disease; RDD, Rosai-Dorfman disease; ALK + H, ALK-positive histiocytosis; HS, histiocytic sarcoma; BPDCN, blastic plasmacytoid dendritic cell neoplasm; FDCS, follicular dendritic cell sarcoma; EBV, Epstein Barr virus; EBV + FDCS, EBV-positive inflammatory follicular dendritic cell sarcoma

Immunohistochemistry (IHC) is a vital tool for the diagnosis of histiocytic and dendritic cell neoplasms. Understanding and interpreting the IHC profiles of each subtype is essential for accurate identification and classification. The following text provides a comprehensive overview of the IHC profiles associated with the different subtypes of these neoplasms. Representative images of immunohistochemical expression for each subtype are displayed in Fig. 5.

### Langerhans cell and other dendritic cell neoplasms

#### Langerhans cell histiocytosis

LCH cells are typically positive for CD1a and show membranous staining. They also express the S100 protein in both the nucleus and cytoplasm and CD207/langerin, which presents as granular cytoplasmic staining. For differential LCH from other types such as indeterminate dendritic cell tumors, immunostaining for CD1a and CD207 should be included. In particular, CD207/langerin immunohistochemical staining is used as a surrogate marker for Birbeck granules, which are the ultrastructural hallmarks of LCH and are observed as cytoplasmic structures in electron microscopy. CD68 often exhibits Golgi dot-like staining. The Ki-67 proliferation index typically varies with indices of < 10%. BRAF-VE1, indicating *BRAF* V600E mutation, may be positive, and both cyclin D1 and p-ERK may be expressed in LCH cells as downstream markers of MAPK activation [1–3, 5, 45–49].

#### Langerhans cell sarcoma

LCS displayed markers similar to those of LCH, including positivity for CD1a, S100, and CD207/langerin. These markers may exhibit focal expression, and the interpretation of immunohistochemistry should focus on the high-grade cells [1–3, 5, 45].

#### Indeterminate dendritic cell tumor

The tumor typically shows positivity for S-100 protein and CD1a but is negative for CD207/langerin [1–3, 5, 29, 30].

#### Interdigitating dendritic cell sarcoma

The tumor cells in this sarcoma were positive for S-100 protein, which highlights dendritic cell processes. Additionally, they express one or more hematolymphoid markers, including CD45, CD4, and CD43. Tumor cells also express fascin, CD68, and lysozyme. They are negative for Langerhans cell markers such as CD1a and langerin, follicular dendritic cell markers like CD21, CD23, and CD35, as well as other markers specific to certain neoplasms or melanoma [1–3, 31, 45].

### Histiocyte/macrophage neoplasms

#### Juvenile xanthogranuloma

JXG cells are typically positive for CD68, CD163, CD4, CD14, factor XIIIa, and fascin. The expression of factor XIIIa and fascin may indicate the origin of dermal macrophages. In a minority of cases, S100 protein may be expressed. CD1a, CD207/langerin, and ALK are negative in JXG. If CD163-positive macrophages show the BRAF VE1 mutation, adult patients should be evaluated for ECD. In children with *BRAF* V600E mutant LCH, this could indicate a lineage switch related to the primary disease [1–3, 6].

#### Erdheim-Chester disease

Cells in ECD typically express CD163, CD68, CD14, factor XIIIa, and fascin, and less commonly express S100 protein. These cells are negative for CD1a and CD207/langerin but may express p-ERK. Diffuse, strong cytoplasmic expression of the BRAF-VE1 suggests a *BRAF* V600E mutation, which should be confirmed with molecular studies [1–4, 6, 50].

#### Rosai–Dorfman disease

RDD is characterized by cells positive for S100 protein, highlighting emperipolesis. The expressions of OCT2 and cyclin D1 are useful for diagnosis. The histiocytic markers CD68 and CD163 were also expressed. Tumor cells typically lack CD1a, CD207/langerin, and ALK. The expression of the *BRAF* V600E mutated protein is rare. For the differential diagnosis of RDD associated with IgG4-related disease, immunostaining for both IgG4 and IgG may be necessary [3, 5, 35, 51, 52].

#### ALK-positive histiocytosis

Under this condition, the tumor histiocytes are, by definition, positive with a cytoplasmic pattern and rarely exhibit membranous or Golgi dot patterns, avoiding nuclear patterns. ALK expression may be weak or focal. At least two histiocytic markers, namely CD68, CD163, CD4, CD14, and lysozyme, are typically expressed. Other markers, such as fascin, factor XIIIa, S100 protein, cyclin D1, and OCT2, may also be positive. These cells usually lack CD1a, CD207/langerin, CD30, and the *BRAF* V600E mutated protein [3, 37].

#### Histiocytic sarcoma

Histiocytic sarcoma cells are positive for two or more histiocytic markers such as CD163, CD68, and lysozyme. S-100 protein may be expressed in a focal, patchy manner. They are negative for CD1a, CD207/langerin (Langerhans cell markers), CD21, CD35 (follicular dendritic cell markers), ALK, and a range of myeloid, melanocytic, epithelial, vascular, and specific mature B-/T- cell markers [1–3, 45].

### Plasmacytoid dendritic cell neoplasms

#### Mature plasmacytoid dendritic cell proliferation associated with myeloid neoplasm

In flow cytometry, MPDCP is detected when cells constitute ≥ 2% of non-erythroid nucleated cells in bone marrow and/or peripheral blood. MPDCP cells show positivity for CD123 (interleukin-3 receptor α-chain) and/or other mature plasmacytoid dendritic cell markers, such as TCF4, TCL1, CD303, and CD304, although aberrant loss of these markers may be detected. Additionally, aberrant expression of CD34, CD56, and TdT was observed in MPDCP cells. Notably, CD56 may be absent, expressed at low levels, or partially expressed. The Ki-67 proliferation index is usually low, particularly when MPDCP is associated with chronic myelomonocytic leukemia [3, 39, 40, 53, 54].

#### Blastic plasmacytoid dendritic cell neoplasm

This neoplasm expresses CD123 and at least one other plasmacytoid dendritic cell marker such as TCF4, TCL1, CD303, or CD304, in addition to CD4 and/or CD56. Alternatively, it may express any of the three plasmacytoid dendritic cell markers in the absence of the expected negative markers of the lymphoid or myeloid lineage, such as CD3, CD14, CD19, CD34, lysozyme, and myeloperoxidase. CD34 is typically negative, and the Ki-67 proliferation index is high [1–3, 54–57].

### Mesenchymal dendritic cell neoplasms of lymphoid tissues: Follicular dendritic cell neoplasms

#### Follicular dendritic cell sarcoma

The cells were positive for two or more FDC markers, including CD21, CD23, CD35, clusterin, CXCL13, and D2-40 (podoplanin). They are negative for S100 protein and lymphocyte-specific markers [1–3, 45, 58].

#### EBV-positive inflammatory follicular dendritic cell sarcoma

These cells express FDC markers, such as CD21, CD35, CD23, CXCL13, and D2-40, or, rarely, fibroblastic/myoid markers, such as smooth muscle actin. They are consistently positive for EBV-encoded small RNA (EBER), characterized by an elongated, bland-looking nuclear pattern and/or a large pleomorphic nuclear pattern [2, 3, 59].

#### Fibroblastic reticular cell tumor

The tumor was positive for cytokeratin, actin, and desmin, highlighting delicate cellular processes. It is negative for follicular dendritic cell markers (CD21, CD23, CD35, and CXCL13) and interdigitating dendritic cell markers (S100) [2, 3, 44].

## Molecular characteristics

Histiocytic and dendritic cell neoplasms show a wide range of genetic mutations and molecular characteristics that play crucial roles in their pathogenesis and clinical behavior [4–6].

**Fig. 6:**
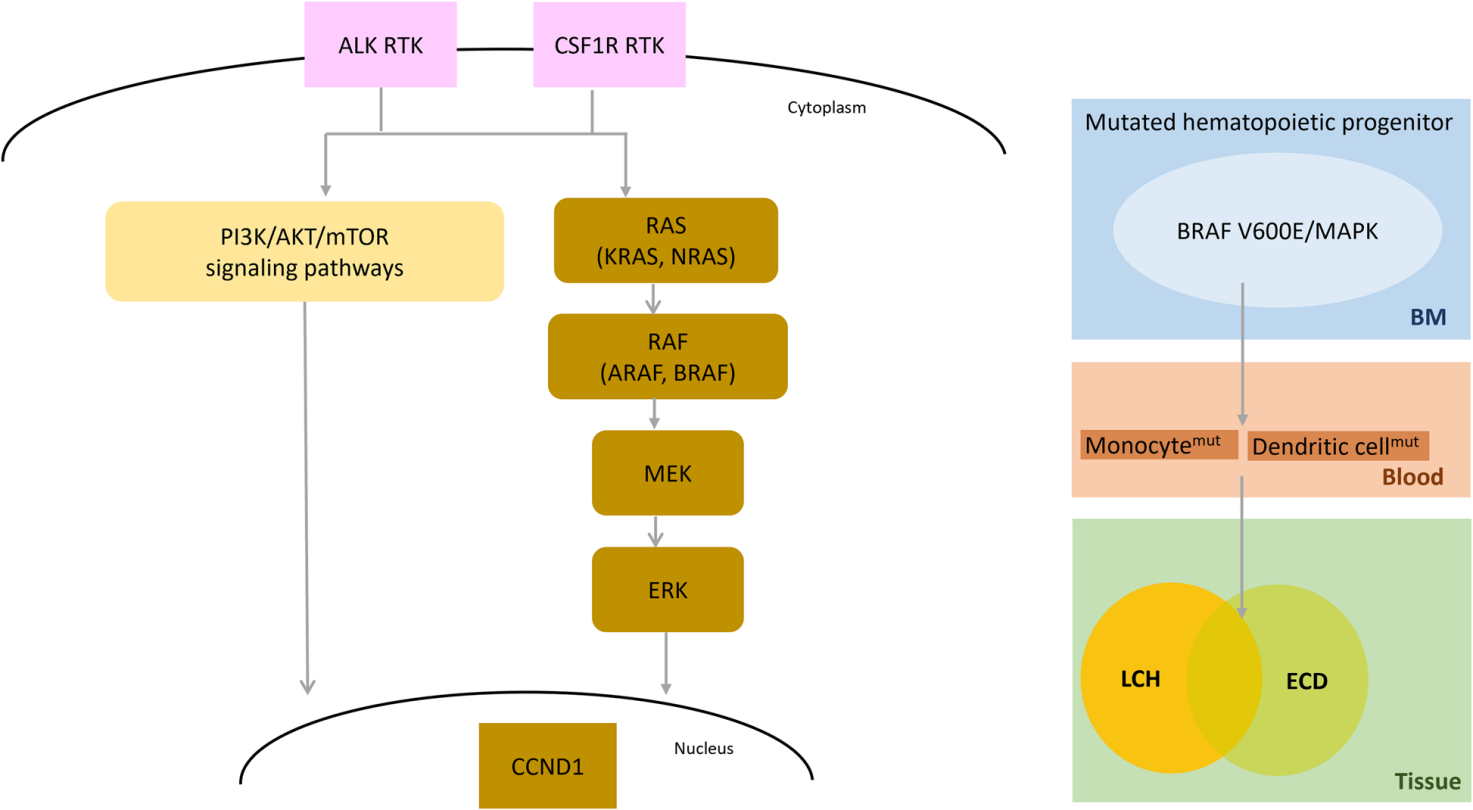
Brief pathways affecting the pathogenesis of histiocytic and dendritic cell neoplasms. The MAPK pathway plays a pivotal role. Mutations in this pathway, especially the *BRAF* V600E mutation, are thought to originate from hematopoietic progenitors in the bone marrow, particularly in the pathogenesis of LCH and ECD. Additionally, gene mutations may occur in elements upstream of *BRAF*, such as *RAS*, or downstream, such as *MAP2K1* (*MEK1*). Moreover, *ALK* gene translocation and *CSF1R* mutation, which affect the transmembrane RTK, can also be present in certain subtypes and are related to both MAPK and PI3K/AKT/mTOR pathways. The proliferation and accumulation of neoplastic histiocytes in tissues can lead to clinical symptoms such as inflammatory reactions, fibrosis, and tissue destruction. Abbreviations: RTK, receptor tyrosine kinase; BM, bone marrow; MAPK, mitogen-activated protein kinase; LCH, Langerhans cell histiocytosis; ECD, Erdheim-Chester disease

Mutations in MAPK pathway genes are frequently observed in various histiocytic and dendritic cell neoplasms. Specifically, the *BRAF* V600E mutation is commonly observed in several subtypes, notably LCH and ECD. Furthermore, both LCH and ECD show mutations in genes of the MAPK pathway in > 80% of cases. Overall, mutations in the MAPK pathway were prevalent, particularly in LCH and ECD within the L group, and were also shared by RDD, JXG, and HS belonging to the R, C, and M groups, respectively. These mutations, particularly the *BRAF* V600E mutation affecting dendritic cell precursors or monocytes, drive the pathological activation of the RAS-RAF-MEK-ERK mitogen-activated protein kinase signaling pathways in diseases such as LCH, ECD, JXG, and RDD (Fig. 6). Through these pathways, mutated histiocytes acquire anti-apoptotic properties and accumulate in the tissues. Subsequently, they release cytokines that recruit inflammatory cells including eosinophils, lymphocytes, and plasma cells. This process leads to tissue inflammation, fibrosis, and eventual destruction of the affected organs or tissues. This highlights the significance of MAPK pathway mutations in the pathogenesis of neoplasms originating from conventional dendritic cells and histiocytes/macrophages [4–6, 60–64].

The following provides an overview of the molecular characteristics associated with diverse types of histiocytic and dendritic cell neoplasms, as well as plasmacytoid dendritic cell neoplasms and follicular dendritic cell neoplasms.

### Langerhans cell and other dendritic cell neoplasms

#### Langerhans cell histiocytosis

LCH is primarily marked by mutations in MAPK pathway genes, most notably *BRAF* V600E as the primary mutation, followed by *MAP2K1*. The MAPK pathway plays a key role in cell division, differentiation, and survival. BRAF is a protein involved in the MAP cell signaling pathway, and *BRAF* V600E mutation leads to constitutive activation. Through the MAP pathway, phosphorylation and nuclear translocation of ERK occur, acting as a transcription factor for cell proliferation and survival. Mutations in MAPK pathway genes are present in approximately 85% of LCH cases, with the *BRAF* V600E mutation occurring in about 50–60% of cases [4, 26, 46, 65–68]. *BRAF* mutations are also correlated with high-risk LCH [46, 69]. *MAP2K1* (or *MEK1*), the downstream kinase of BRAF, mutations are detected in approximately 20% of the cases and occur in a mutually exclusive manner with *BRAF* mutations [68, 70]. *BRAF* mutations can occur at sites other than the V600 residue and may also be found in other RAF kinases, such as *ARAF*. Additionally, alterations can occur in other components of the MAP signaling pathway upstream of BRAF, including *NRAS, KRAS*, and *HRAS*. These somatic activating mutations in the genes of the MAPK pathway have been detected in a mutually exclusive manner [4, 65, 71].

The “misguided myeloid differentiation model” for LCH ontogeny, which is associated with MAPK activation, proposes a differentiation in the origin of LCH based on the level of risk and systemic involvement. According to this model, high-risk multisystem LCH (multisystem with risk-organ involvement, MS-RO +) is believed to originate from driver mutations in multipotent hematopoietic progenitor cells existing in the bone marrow. These cells can differentiate into various myeloid cells, including dendritic cells and monocytes/macrophages. In contrast, low-risk LCH, both multisystem without risk organ involvement (MS-RO-) and single-system LCH, is thought to arise from driver mutations in circulating or tissue-resident DC-committed precursors [46, 72]. The *BRAF* V600E mutation plays a crucial role in LCH pathophysiology by disrupting cell migration, inhibiting apoptosis, and inducing oncogene-related senescence [3, 5, 66, 73, 74].

#### Langerhans cell sarcoma

A subset of these cases shows mutations in the MAPK pathway, including alterations in the *KRAS* gene and, less frequently, the *BRAF* V600E mutation. In secondary LCS, clonality or molecular alterations may be shared with the original hematologic neoplasm [2, 3, 27, 28, 75].

#### Indeterminate dendritic cell tumor and interdigitating dendritic cell sarcoma

Due to the rarity of indeterminate dendritic cell tumors and interdigitating dendritic cell sarcomas, comprehensive information specific to these tumors is limited. In indeterminate dendritic cell tumor, mutations in genes such as *BRAF* V600E are reported, albeit rarely [63]. Interdigitating dendritic cell sarcoma, on the other hand, is characterized by mutations in the MAPK pathway, including *BRAF* V600E, *KRAS, NRAS*, and *MAP2K1* [63, 75].

### Histiocyte/macrophage neoplasms

#### Juvenile xanthogranuloma

JXG is characterized by alterations in the MAPK pathway, including activating mutations in *MAPK21, NRAS, KRAS, CSF1R, BRAF*, and *NTRK1* fusions [3, 6, 68, 76].

#### Erdheim-Chester disease

ECD is characterized by mutations in several MAPK pathway genes, including *BRAF* V600E, *ARAF, NRAS, KRAS, MAP2K1*, and *PIK3CA*. The *BRAF* V600E mutation occurs in approximately 50–60% of ECD cases. Mutations in MAPK pathway genes, such as *BRAF* V600E and *MAP2K1*, have been detected in bone marrow progenitors, indicating that ECD ontogeny is associated with MAPK activation. The presence of these mutations in early progenitor cells highlights the significant role of MAPK pathway activation in ECD pathogenesis. Furthermore, mutations in other genes of the MAPK pathway, such as *MAP2K1, ARAF, NRAS,* and *KRAS*, have been observed in ECD, as well as in *PIK3CA*, which is part of the PI3K-AKT-activating pathway. Regarding prognosis, the implications of *BRAF* mutations in ECD have not shown differences from other alternative mutations, although *BRAF* mutations are associated with an increased risk of cardiovascular involvement [4–6, 60–62, 77, 78].

#### Rosai-Dorfman disease

Although the mutation frequency is much lower than that in LCH, JXG, and ECD, RDD involves mutations in the MAPK/ERK pathway, encompassing genes such as *KRAS, NRAS, MAP2K1, ARAF, CSF1R,* and on rare occasions, *BRAF* V600E [6, 52, 62, 76].

#### ALK-positive histiocytosis

ALK-positive histiocytosis is characterized by rearrangements in the ALK gene, with the *ALK::KIF5B* fusion being more commonly detected. Fusions with uncommon partners such as *CLTC-ALK, TPM3-ALK, TFG-ALK, EML4-ALK*, and *DCTN1-ALK* have also been identified. These *ALK* rearrangements lead to the activation of ALK kinase, which in turn activates several downstream signaling pathways crucial for cell growth and survival, including the MAPK pathway [33, 37].

#### Histiocytic sarcoma

Mutations commonly occur in the MAPK pathway in histiocytic sarcomas. These mutations often involve genes such as *KRAS*, *BRAF* V600E, *NRAS, MAP2K1*, and *CSF1R* [76, 79].

### Plasmacytoid dendritic cell neoplasms

#### Mature plasmacytoid dendritic cell proliferation associated with myeloid neoplasm

When associated with myeloid neoplasms, such as CMML, clonal tumor cells from MPDCP often exhibit mutations in the RAS pathway. In cases associated with AML, clonal MPDCP cells share a mutational landscape similar to that of CD34 + blasts [38, 40].

#### Blastic plasmacytoid dendritic cell neoplasm

Blastic plasmacytoid dendritic cell neoplasm is characterized by the activation of the NF-κB pathway [80]. The E-box transcription factor TCF4 (known as transcription factor E2-2) is a critical regulator of BPDCN development [81].

### Mesenchymal dendritic cell neoplasms of lymphoid tissues: Follicular dendritic cell neoplasms

Follicular dendritic cell sarcoma recurrently shows alterations in the NF-kB pathway and *BRAF* V600E mutations [82, 83]. Detailed molecular characteristics of EBV-positive inflammatory follicular dendritic cell sarcomas and fibroblastic reticular cell tumors remain limited owing to the rarity of these conditions.

## Conclusion

This comprehensive review article presents a detailed analysis of the ontogeny, morphology, immunophenotypic features, and molecular characteristics of histiocytic and dendritic cell neoplasms. Various categories and subtypes of these neoplasms have been investigated, highlighting their cellular origins, typical immunophenotypes, and neoplastic counterparts.

Morphologically, these neoplasms exhibit diverse histopathological features that are critical for the diagnosis. From the large round histiocytes in LCH to the spindle-shaped cells in FDCS, each subtype presented unique characteristics. Immunophenotypic analysis, primarily immunohistochemistry, plays a pivotal role in classification. Markers such as CD1a, S100, and CD207 for LCH, and other markers such as CD68 and CD163 for histiocyte/macrophage neoplasms have been discussed in detail. The molecular landscape of these neoplasms is marked by a variety of genetic mutations, particularly in the MAPK pathway genes. Mutations, such as *BRAF* V600E, in LCH and ECD have been highlighted. These molecular insights offer potential avenues for the development of targeted therapies.

**Fig. 7:**
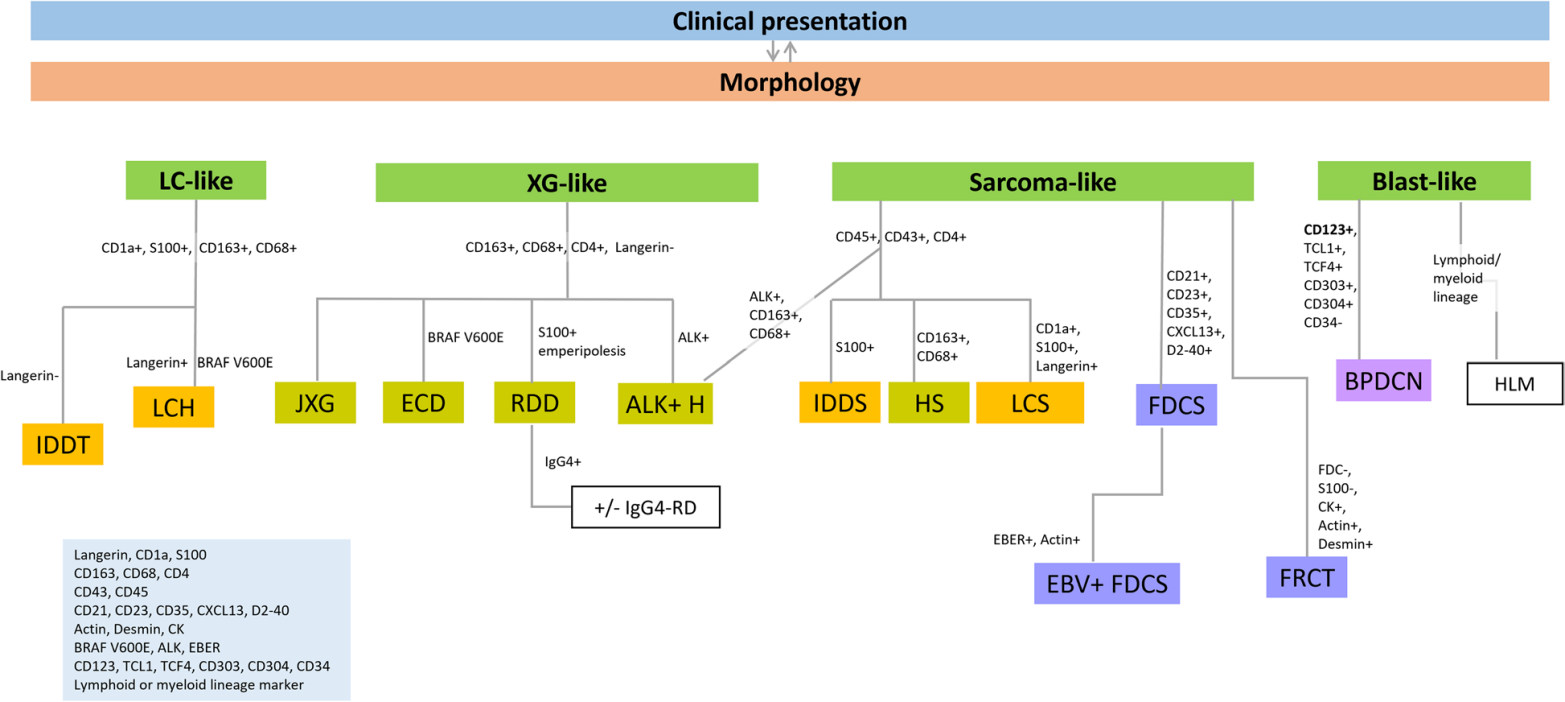
Overall diagnostic process flow for the classification of histiocytic and dendritic cell neoplasms incorporates clinical presentations, morphology, immunohistochemistry, and molecular markers. Abbreviations: LC, Langerhans cell; XG, xanthogranuloma; IDDT, indeterminate dendritic cell tumor; LCH, Langerhans cell histiocytosis; JXG, juvenile xanthogranuloma; ECD, Erdheim-Chester disease; RDD, Rosai-Dorfman disease; IgG4-RD, IgG4-related disease; ALK + H, ALK-positive histiocytosis; IDDS, interdigitating dendritic cell sarcoma; HS, histiocytic sarcoma; LCS, Langerhans cell sarcoma; FDCS, follicular dendritic cell sarcoma; EBV, Epstein Barr virus; EBV + FDCS, EBV-positive inflammatory follicular dendritic cell sarcoma; FRCT, fibroblastic reticular cell tumor; BPDCN, blastic plasmacytoid dendritic cell neoplasm; HLM, hematolymphoid malignancies

Briefly, these tumors can be morphologically categorized as Langerhans cell, xanthogranuloma, sarcoma/spindle cell, or blast-like. Immunohistochemical markers for Langerhans cells (CD1a, S100, and langerin), histiocytes/macrophages (CD68, CD163, and CD4), hematolymphoid markers (CD43 and CD45), FDC markers (CD21, CD23, CD35, CXCL13, and D2-40), FRC markers (actin and desmin), and pDC markers (CD123, TCL1, TCF4, CD303, and CD304), along with specific molecular or viral markers (*BRAF* V600E, ALK, and EBER), are employed in a step-wise diagnostic approach. When it is necessary to differentiate them from other subtypes, lymphoid and myeloid lineage markers may be instrumental in excluding specific subtypes of hematolymphoid malignancies. Figure 7 provides an overview of the diagnostic process.

Overall, our understanding of histiocytic and dendritic cell neoplasms has evolved significantly, encompassing the intricate details of their cellular origins, morphological diversity, immunophenotypic profiles, and molecular underpinnings. This knowledge is crucial for the accurate diagnosis and development of effective treatment strategies for these rare but complex tumors.

## Data Availability

No datasets were generated or analysed during the current study.

## Abbreviations

MPS: Mononuclear phagocyte system
HSCs: Hematopoietic stem cells
CMPs: Common myeloid progenitors
GMPs: Granulocyte–macrophage progenitors
cMoPs: Common monocyte progenitors
pDC: Plasmacytoid dendritic cell
cDC: Conventional dendritic cell
LCH: Langerhans cell histiocytosis
LC: Langerhans cells
TME: Tumor microenvironment
LCS: Langerhans cell sarcoma
MAPK: Mitogen-activated protein kinase
IDDT: Indeterminate dendritic cell tumor
IDDS: Interdigitating dendritic cell sarcoma
JXG: Juvenile xanthogranuloma
ECD: Erdheim-Chester disease
RDD: Rosai-Dorfman disease
ALK + H: ALK-positive histiocytosis
HS: Histiocytic sarcoma
MPDCP: Mature plasmacytoid dendritic cell proliferation associated with myeloid neoplasm
BPDCN: Blastic plasmacytoid dendritic cell neoplasm
FDCS: Follicular dendritic cell sarcoma
EBV: Epstein Barr virus
FRCT: Fibroblastic reticular cell tumor
